# Chaperones in Polyglutamine Aggregation: Beyond the Q-Stretch

**DOI:** 10.3389/fnins.2017.00145

**Published:** 2017-03-23

**Authors:** E. F. E. Kuiper, Eduardo P. de Mattos, Laura B. Jardim, Harm H. Kampinga, Steven Bergink

**Affiliations:** ^1^Department of Cell Biology, University Medical Center Groningen, University of GroningenGroningen, Netherlands; ^2^Programa de Pós-Graduação em Genética e Biologia Molecular, Department of Genetics, Universidade Federal do Rio Grande do SulPorto Alegre, Brazil; ^3^Medical Genetics Service, Hospital de Clínicas de Porto AlegrePorto Alegre, Brazil; ^4^Departamento de Medicina Interna, Universidade Federal do Rio Grande do SulPorto Alegre, Brazil

**Keywords:** aggregation, Huntington's disease, Machado-Joseph disease, molecular chaperones, polyglutamine disease

## Abstract

Expanded polyglutamine (polyQ) stretches in at least nine unrelated proteins lead to inherited neuronal dysfunction and degeneration. The expansion size in all diseases correlates with age at onset (AO) of disease and with polyQ protein aggregation, indicating that the expanded polyQ stretch is the main driving force for the disease onset. Interestingly, there is marked interpatient variability in expansion thresholds for a given disease. Between different polyQ diseases the repeat length vs. AO also indicates the existence of modulatory effects on aggregation of the upstream and downstream amino acid sequences flanking the Q expansion. This can be either due to intrinsic modulation of aggregation by the flanking regions, or due to differential interaction with other proteins, such as the components of the cellular protein quality control network. Indeed, several lines of evidence suggest that molecular chaperones have impact on the handling of different polyQ proteins. Here, we review factors differentially influencing polyQ aggregation: the Q-stretch itself, modulatory flanking sequences, interaction partners, cleavage of polyQ-containing proteins, and post-translational modifications, with a special focus on the role of molecular chaperones. By discussing typical examples of how these factors influence aggregation, we provide more insight on the variability of AO between different diseases as well as within the same polyQ disorder, on the molecular level.

## Introduction

**Figure 1:**
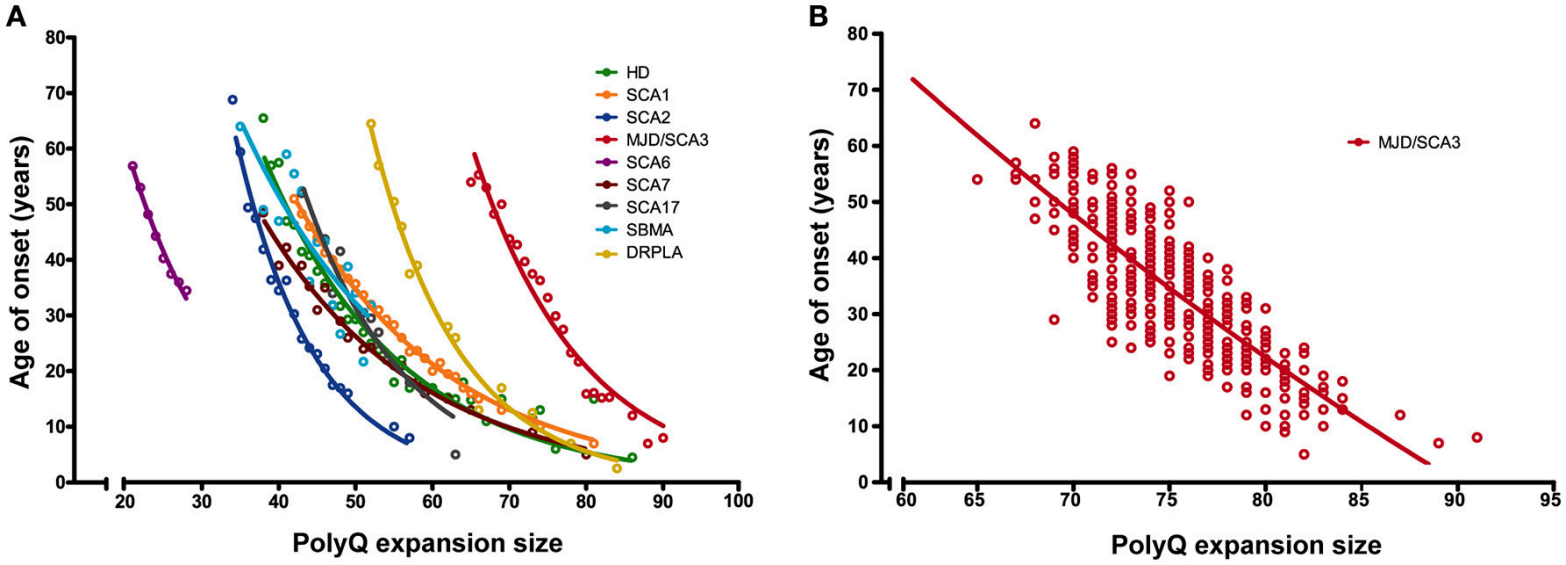
**Age of onset of disease inversely correlates with the size of the expanded polyQ tract in all known polyQ diseases. (A)** Correlation between age of onset (AO) and CAG expansion size for all nine polyQ diseases identified so far. Circles depict mean AOs for a given expansion size based on multiple reported cohorts of patients. Lines represent the fitted data according to an exponential decay model. **(B)** Age of onset of disease is not completely determined by the expanded polyQ tract alone. Data on the variability of AO for a particular polyQ expansion size is shown as in **(A)** and was based on the large cohort of MJD/SCA3 patients reported by Saute and Jardim (2015). Circles represent single patients. Please refer to Supplementary File 1 for a complete list of references of the original cohort descriptions. Note that graph **(A,B)** are not drawn to the same scale.

Polyglutaminopathies are a family of diseases characterized by CAG trinucleotide expansions in the coding regions of at least nine unrelated genes, resulting in proteins with an abnormally long polyglutamine (polyQ) stretch, which have a high aggregation propensity. PolyQ aggregates can impede cellular protein homeostasis, loss of which is also observed in many other neurodegenerative diseases (Soto, 2003). These mutant proteins lead to one recessive inherited, X-linked spinal and bulbar muscular atrophy (SBMA), and eight dominantly inherited neuronal dysfunctions, Huntington's disease (HD), dentatorubral pallidoluysian atrophy (DRPLA), and the spinocerebellar ataxias (SCAs) type 1, 2, 3, 6, 7, and 17 (Margolis and Ross, 2001). All known polyglutaminopathies show a strong inverse correlation between expansion size and age at onset (AO) of the disease, with longer repeats significantly correlating with earlier onset of symptoms and higher aggregation proneness of the affected protein, indicating that an expanded polyQ is tightly related to the diseases. There are two main features that are striking in the association between polyQ length and AO. First, there is marked variability between polyQ diseases in expansion thresholds that determines the pathogenicity, indicating that AO has only a partial dependence on the polyQ stretches and their absolute lengths (Figure 1A). Second, there is also CAG-length independent phenotypic variation within a given polyQ disease (Figure 1B). Both these findings imply that factors beyond the polyQ stretch are co-determining disease onset (Ranum et al., 1994; DeStefano et al., 1996; Hayes et al., 2000; Wexler et al., 2004; van de Warrenburg et al., 2005; Kaltenbach et al., 2007; Branco et al., 2008; Lessing and Bonini, 2008; Bettencourt et al., 2011; Tezenas du Montcel et al., 2014; Bečanović et al., 2015). It was hypothesized that the differential effects of distinct polyQ proteins with polyQ tracts of similar lengths could be, at least in part, due to the sequences flanking the polyQ expansion (Nozaki et al., 2001).

**Figure 2:**
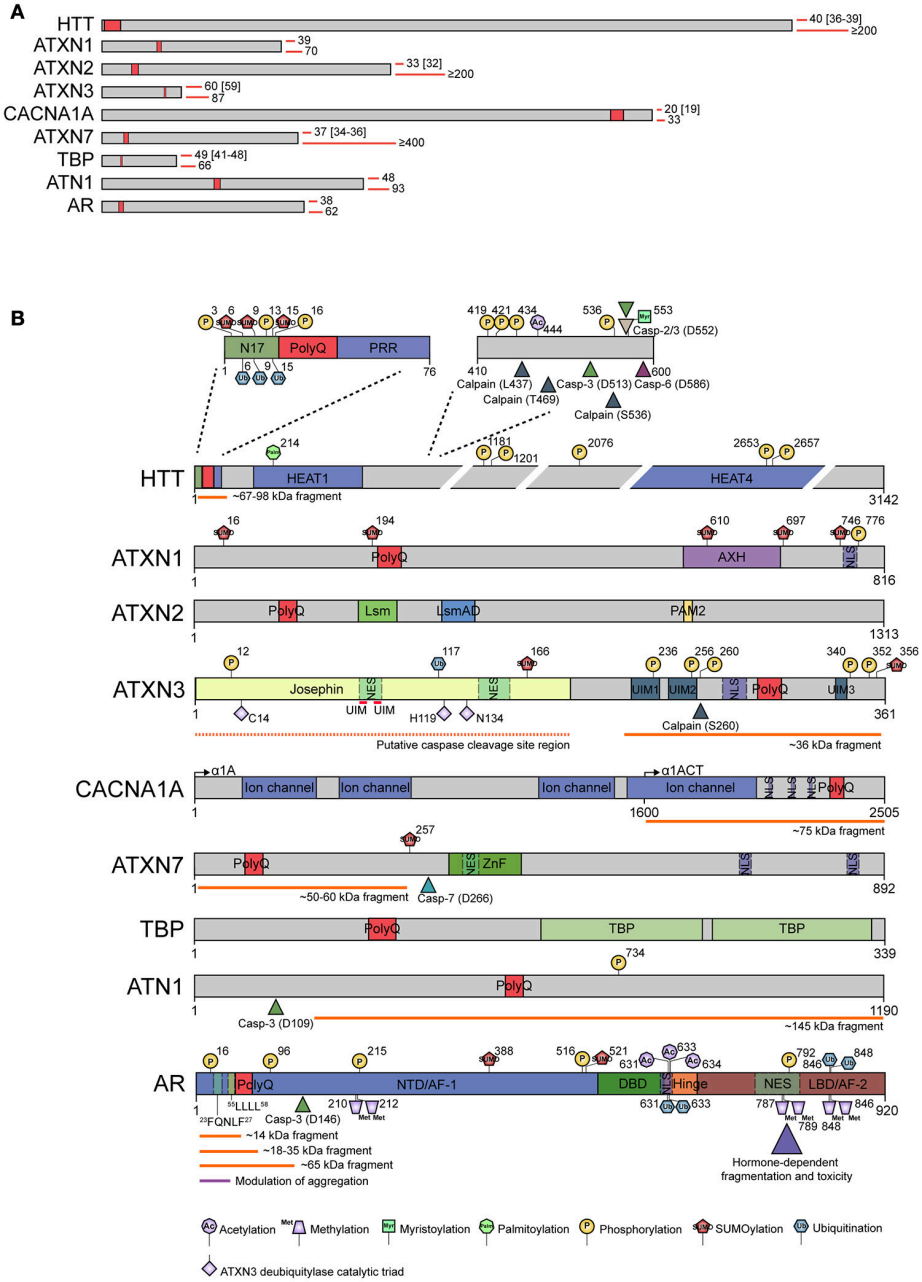
**Representation of pathogenic polyQ proteins and known modulating events associated with aggregation. (A)** Schematic representation of the nine disease-related polyglutamine proteins drawn to scale. In each case, a polyQ stretch of fixed length is depicted at the approximate position (red boxes). Red bars on the right side of each protein show the smallest and largest number of glutamine repeats identified in patients of each polyQ disease to date. Numbers between brackets represent polyQ expansion sizes that have been reported to behave as incomplete penetrance alleles. **(B)** Detailed representation of all nine polyQ proteins. Domain organization is indicated. Known post-translational modifications associated with disease, caspase/calpain cleavage sites, and fragments identified are indicated. For ataxin-3, the long isoform with 3 ubiquitin-interacting motifs is shown. Residues C14, H119, and N134 depict the catalytic triad of the deubiquitylase activity of the Josephin domain. The CACNA1A locus encodes two proteins: α1A (full-length α1A) and α1ACT (C-terminal fragment of α1A) using a bicistronic mRNA with a cryptic internal ribosomal entry site. The polyQ is found in both. Many studies report a C-terminal fragment which probably represents α1ACT. For the androgen receptor, the only phosphorylation sites depicted are those with biochemical evidence of modulation of polyQ aggregation, cleavage and/or toxicity. Similarly, amino acid sequences 23FQNLF27 and 55LLLL58 highlight motifs shown to influence polyQ behavior. For simplicity, most huntingtin cleavage products are omitted and only the major N-terminal polyQ containing fragment is indicated. Amino acid numbering is based on Uniprot accession numbers P42858 (HTT), P54253 (ATXN1), Q99700 (ATXN2), P54252 (ATXN3), O00555 (CACNA1A), O15265 (ATXN7), P20226 (TBP), P54259 (ATN1), and P10275 (AR). However, for clarity, some residues are numbered according to their original publication, which might differ from the numbering according to the reference protein sequence (due to the expanding nature of polyQ proteins). AR, androgen receptor; ATN1, atrophin-1; ATXN1, ataxin-1; ATXN2, ataxin-2; ATXN3, ataxin-3; ATXN7, ataxin-7; AXH, ataxin-1/high-mobility group box containing protein-1; CACNA1, α1A subunit of the P/Q-type or CaV2.1 voltage-gated calcium channel; Casp, caspase; DBD, DNA binding domain; HTT, huntingtin; PolyQ, polyglutamine stretch; NTD/AF-1, amino-terminal domain/ activation function-1; LBD/AF-2, ligand-binding domain/ activation function-2; NES, nuclear export signal; NLS, nuclear localization signal; HEAT, huntingtin/elongation factor 3/PR65/A subunit of protein phosphatase 2A/ lipid kinase TOR domain; PRR, proline-rich region; N17, first 17 amino acids of huntingtin; TBP, TATA-binding protein (domain); UIM, ubiquitin-interacting motif; Ub-1/Ub2, ubiquitin-binding sites; Lsm, Like RNA splicing domain Sm and Sm2; LsmAD, Like-Sm-associated domain; PAM2, poly (A)-binding protein interacting motif 2; ZnF, SCA7-like zinc finger domain. For references to specific domains or post-translational modifications, please refer to Supplementary File 1.

Here we discuss that, next to aggregation of the core polyQ stretch, which is common to all polyglutaminopathies (Figure 2A), the context around the cores can modulate aggregation in several ways and may be linked to differential handling of the protein quality control systems, including molecular chaperones, the ubiquitin proteasome system, and autophagy. These degradation processes, and their relationship with the chaperone system, are of importance and greatly influence the aggregation process (Rubinsztein, 2006). Certain chaperones act together with the protein degradation machineries to effectively clear aggregation-prone polypeptides, such as polyQ-containing proteins (Dekker et al., 2015). The molecular details of these downstream events are still unclear and will not be discussed here; instead we will focus on the impact of molecular chaperones on the aggregation process itself. Molecular chaperones are known to influence aggregation of polyQ proteins. This could either be directly by preventing the polyQ stretch from aggregating or via the flanking sequences. For only a few of the molecular chaperones the direct interaction with the polyQ proteins has been shown, although many chaperones are found to co-localize with polyQ inclusions (Cummings et al., 1998; Kazemi-Esfarjani and Benzer, 2000; Schmidt et al., 2002; Helmlinger et al., 2004; Bilen and Bonini, 2007; Hageman et al., 2010; Gao et al., 2011; Kakkar et al., 2014; Matilla-Dueñas et al., 2014; Reis et al., 2016; Zhao et al., 2016). However, co-localization of chaperones does not provide information on their mode of interaction and does not distinguish whether chaperones are truly interacting with the polyQ protein, or whether the presence of chaperones in the aggregates is a mere secondary effect due to a collapse of other cellular components with the inclusions. In this review, we will discuss: first, how polyQ tracts drive aggregation; second, how their flanking sequences could directly affect the aggregation proneness of the polyQ protein; and third, how polyQ proteins can be modified, changed in conformation, or fragmented, inducing aggregation (Figure 2B). We will not focus on the function, or loss of function, of the affected polyQ proteins, since this was so far not shown to be causative for disease, even though the native function of the protein might be important for normal cellular function. Furthermore, we will not go into the discussion on the toxicity of aggregation. For instance, it is still unclear whether the presence of aggregates contributes to SCA2 pathology (Huynh et al., 2000), even though aggregates are found in affected brain areas (Pang et al., 2002; Seidel et al., 2016). Finally, we will highlight the role of chaperones in the aggregation process and include only studies that provide insight in direct interaction of chaperones with the polyQ proteins. Rather than providing a complete overview, molecular mechanisms of typical examples will be discussed, aiming at providing general principles affecting polyQ aggregation on the molecular level that may partially explain the individual differences between patients and steer future studies.

## Aggregation properties of the polyQ stretch

Aggregates formed by polyQ stretches contain identical β-strand-based cores. Already in 1994, Perutz et al. described the ability of elongated polyQ stretches to form β-sheets (Perutz et al., 1994). Like many other amyloidogenic proteins (Sawaya et al., 2007), the polyQ chains can form β-sheets that are connected through interdigitating extended side chains and contain intramolecular β-hairpins (Hoop et al., 2016). Formation of β-hairpins allows for hydrogen bonding between the stacked side chains, providing a strong interaction (Hoop et al., 2016). The β-hairpins play an important role in the aggregation process. Q-stretches with a range up to 25Q are not able to form stable β-hairpins and therefore are not able to induce aggregation, except when mutations known to enhance β-hairpin formation are introduced (Kar et al., 2011, 2013). It is hypothesized that longer polyQ stretches can form more stable intramolecular β-hairpins, providing a critical monomeric nucleus necessary for inducing aggregation (Kar et al., 2011). The high affinity of the β-sheets affects interactions between molecules and might not only do so for the same pathogenic polyQ protein, but also as a secondary effect for other endogenous polyQ containing proteins (Nóbrega et al., 2015). For example, the endogenous, non-expanded TATA-box binding protein (TBP) was found to sequester into aggregates formed by other pathogenic polyQ proteins, such as huntingtin (HTT; Perez et al., 1998; Kim et al., 2002; Matsumoto et al., 2006). Similarly, inclusions containing ataxin-2 (ATXN2), ataxin-3 (ATXN3) and TBP are observed in SCA1, SCA2, SCA3, and DRPLA (Uchihara et al., 2001). Whether these secondary co-aggregating events contribute to disease is currently not clear (Kampinga and Bergink, 2016).

The crucial role for the formation of β-hairpins in the aggregation process is nicely illustrated by findings on missense CAG to CAT mutations. These mutations, coding for histidine, were found in the CAG-repeat in ATXN1, leading to insertion of one or more other amino acids and interrupting the Q-stretch (Sobczak and Krzyzosiak, 2004; Jayaraman et al., 2009; Menon et al., 2013). The AO is in these cases inversely correlated to the longer uninterrupted CAG stretch which, rather than a specific interruption pattern, dictates also the aggregation propensity *in vitro* (Menon et al., 2013). The structure of the polyQ-stretches is not changed because of the histidine-interruptions but the polyQ aggregation rates are decreased due to the Q-length dependent ability of the protein to form a critical nucleus to initiate aggregation (Jayaraman et al., 2009; Menon et al., 2013).

From all the different intracellular chaperones, so far the only ones described that could act on the β-sheets or β-hairpins formed by the Q-stretch are DNAJB6 and its closest homolog DNAJB8, two members of the DNAJ family of Hsp70 co-chaperones. In a screen for suppressors of aggregation of huntingtin (HTT-119Q) both DNAJB6 and DNAJB8 were superior suppressors of aggregation with a specificity for the polyQ tract, since they were similarly effective in the suppression of aggregation of HTT, ATXN3, the androgen receptor (AR), and polyQ alone (Hageman et al., 2010; Månsson et al., 2013). These DNAJ chaperones have a unique region containing 18 residues of the polar hydroxyl group amino acids serine and threonine, that is exposed on one face of the DNAJB6 monomer where it is predicted to interact with the hydrogen bonds in the polyQ β-hairpins (Månsson et al., 2013; Kakkar et al., 2016).

## Aggregation initiation by flanking domains in polyQ-containing proteins

A longer Q-stretch not only has a higher aggregation propensity, but also affects the conformation of other parts of the protein. This can cause exposure of other regions in the proteins that have aggregation-prone properties by themselves (Ellisdon et al., 2006; Kelley et al., 2009; Tam et al., 2009). The intrinsic aggregation propensity leads to a two-stage aggregation mechanism (Ellisdon et al., 2006) in which the first aggregation step is actually thought to be a nucleation step of the non-polyQ-containing flanking domains. The formed nucleus can speed up the aggregation of the polyQ-stretch, which is then the second aggregation step. Aggregation of the flanking region and the polyQ stretch may enhance each other in a positive feedback loop accelerating aggregation and AO (Ellisdon et al., 2007; Saunders et al., 2011). The most striking examples of this process are known for HTT and ATXN3.

HTT is a relatively large protein with the polyQ stretch located in the first exon of the protein. The polyQ tract in HTT is flanked by a 17 amino acid long N-terminal (N17) domain and a polyproline domain on its C-terminus (Dehay and Bertolotti, 2006; Rockabrand et al., 2007; Figure 2). The N17 domain is highly soluble by itself and has an intrinsic tendency to collapse into an aggregation-resistant compact coil state (Thakur et al., 2009; Crick et al., 2013). When the Q-stretch is expanded, the N17 domain undergoes a conformational change going into a more α-helical extended state (Tam et al., 2009; Thakur et al., 2009; Sivanandam et al., 2011), exposing a hydrophobic face through which self-association is induced (Kelley et al., 2009; Liebman and Meredith, 2010). Self-association provides an initial nucleus that increases the local concentration of the adjacent polyQ, promoting polyQ aggregation (Kelley et al., 2009; Liebman and Meredith, 2010; Sahoo et al., 2016). Aggregation of HTT can be prevented by modifying the hydrophobic face of the α-helix (Tam et al., 2009), confirming the important role of the N17 domain in initial aggregation. Moreover, synthetic polyQ peptides lacking the N17 domain show much slower aggregation kinetics (Månsson et al., 2013; Monsellier et al., 2015; Sahoo et al., 2016).

The exposed hydrophobic face on the N17 domain was identified as an interaction site for several chaperones amongst which the chaperonin TRiC, specifically the subunit CCT1 (Tam et al., 2006). CCT1 can suppress HTT aggregation by binding via its apical substrate-binding domains to the hydrophobic motifs in the N17, preventing the initial step of aggregation (Spiess et al., 2006; Tam et al., 2009; Shahmoradian et al., 2013; Sahl et al., 2015). The constitutively expressed Hsp70 (Hsc70/HSPA8) was found to co-localize, like many other Hsp70s including the prokaryotic DnaK and yeast Ssa1 (Jana et al., 2000; Muchowski et al., 2000; Novoselova et al., 2005; Tam et al., 2006), and interact with the N17 domain of HTT via its client protein binding domain (Monsellier et al., 2015). HSPA8 is not able to delay aggregation of a Q-stretch lacking flanking sequences (Månsson et al., 2013) and acts, similar to CCT1, by disrupting the interaction between N17 domains of HTT, slowing down aggregate formation (Monsellier et al., 2015).

Another example of a polyQ protein that undergoes a similar two-stage aggregation mechanism is ATXN3, causative for SCA3. ATXN3 is involved in proteostasis by editing specific ubiquitin sidechains that are targeting proteins to the proteasome (Kuhlbrodt et al., 2011). ATXN3 has an unstructured C-terminus containing the polyQ expansion and multiple ubiquitin interacting motifs (UIMs), and an N-terminus containing the Josephin domain (JD), which is a structured monomeric domain that folds into a globular conformation (Chow et al., 2004; Masino et al., 2004; Figure 2). The JD is the catalytic domain responsible for the deubiquitinating (DUB) properties of ATXN3 and has a high α-helical content forming a groove with two additional UIMs for recognition of the polyubiquitin chains of different linkages, and positioning them for cleavage (Masino et al., 2004; Nicastro et al., 2009, 2010). Sequence motifs on the helices in the groove are functionally important for binding conjugated ubiquitin but are predicted to be highly amyloidogenic and therefore responsible for the aggregation propensity of the JD itself (Masino et al., 2011; Lupton et al., 2015). Indeed, *in vitro* the isolated JD shows fibrillogenic behavior even under physiological conditions (Masino et al., 2004, 2011; Ellisdon et al., 2006), but when ubiquitin is added, the aggregation propensity of ATXN3 is lowered (Masino et al., 2011). Expansion of the polyQ stretch influences the conformation of the JD in such a way that the molecular mobility of two α-helices is increased and the amyloidogenic motif gets more exposed (Lupton et al., 2015; Scarff et al., 2015), providing a nucleus through which the first aggregation step of ATXN3 is initiated. This can in turn accelerate aggregation of the polyQ stretch (Gales et al., 2005; Ellisdon et al., 2007). In a dedicated screen, several modifiers of ATXN3 were identified that all fell into the canonical chaperone and ubiquitin pathways (Bilen and Bonini, 2007). Amongst the chaperones was alphaB-crystallin (HSPB5), which was found to interact with the JD in the distorted ubiquitin interacting groove, possibly masking the amyloidogenic motives, and having an effect on the initial nucleation step of ATXN3 (Robertson et al., 2010).

Flanking regions can also suppress aggregation of the polyQ stretch. For example, the proline-rich flanking domain (C38) in HTT has an opposite effect compared to the N17 domain. The C38 is also highly soluble, but actually lowers the rate of aggregation (Bhattacharyya et al., 2006; Dehay and Bertolotti, 2006; Duennwald et al., 2006; Crick et al., 2013). Other polyQ-containing proteins apart from HTT, also have a proline-rich region adjacent to the Q-stretch, like TBP, AR, and ATXN2 (Kim, 2014). It is tempting to speculate that these regions confer an evolutionary benefit and co-evolved with Q stretches to modulate their aggregation.

## Binding partners that can influence aggregation

As we have now seen, the opening up of physiologically needed hydrophobic, aggregation-prone, motifs in non-polyQ-containing parts of the protein, can lead to the unwanted formation of an initial nucleus for aggregation. These motifs are normally buried or in interaction with binding partners (or substrates), like ubiquitin in the case of ATXN3, which prevents exposure of the hydrophobic regions (Masino et al., 2011). Binding partners of polyQ-containing proteins can influence the aggregation to a great extent, also for ataxin-1 (ATXN1). ATXN1 is the protein that underlies SCA1, and has a Q-stretch in the N-terminal part of the protein and an AXH domain in the C-terminus (Figure 2). Just like the JD in ATXN3, the AXH domain in ATXN1 has aggregation-prone properties that are needed for its normal functioning, but therefore can be detrimental in the presence of an expanded polyQ stretch (De Chiara et al., 2013a). The AXH domain is responsible for transcriptional repression, RNA-binding activity, and is necessary for interacting with other proteins, mostly transcriptional regulators. For the domain to be able to bind all its different substrates, it has a remarkable conformational plasticity (Chen et al., 2004; De Chiara et al., 2013b; Deriu et al., 2016). Moreover, the AXH domain is responsible for ATXN1 self-association. Multimerization can bring polyQ stretches together, associated with aggregation and amyloid formation (De Chiara et al., 2005b, 2013a; Lasagna-Reeves et al., 2015). *In vivo* ATXN1 forms oligomers and interestingly the interaction partner transcriptional repressor Capicua (CIC) is found in these complexes (Lam et al., 2006; Lasagna-Reeves et al., 2015). The interaction of CIC with the AXH domain of ATXN1 stabilizes toxic soluble prefibrillar oligomers of ATXN1. When CIC levels are reduced, ATXN1 forms more fibrillar oligomers that are less toxic (Lasagna-Reeves et al., 2015). Also when the AXH domain is deleted, aggregate formation is reduced (De Chiara et al., 2005a,b). There are chaperones known to prevent ATXN1 aggregation and reduce toxicity, but the exact mechanism of action of the chaperones on ATXN1 is not known (Cummings et al., 1998; Zhai et al., 2008). A possible mechanism of action could be that chaperones bind to the AXH domain of ATXN1 to prevent complex formation or to prevent CIC from binding.

## Cleavage/fragmentation

Fragmented polyQ proteins have been found in patients and proteolytic processing of polyQ proteins into smaller, highly aggregation-prone fragments that are more toxic than the full-length protein has been described for most polyQ diseases, HD (Mangiarini et al., 1996; Martindale et al., 1998), DRPLA (Igarashi et al., 1998; Wellington et al., 1998), SBMA (Butler et al., 1998; Kobayashi et al., 1998; Wellington et al., 1998), and SCAs (Ikeda et al., 1996; Paulson et al., 1997; Zander et al., 2001; Goti et al., 2004; Helmlinger et al., 2004; Kordasiewicz et al., 2006; Matos et al., 2016a; Figure 2B). However, for SCA1, SCA2, and SCA17 the evidence for the presence of fragments is limited (Matos et al., 2016a). Proteases play a key role in the generation of these polyQ fragments, and inhibition of proteases or mutation of their cleavage sites can modulate the disease AO (Ona et al., 1999; Chen et al., 2000; Graham et al., 2006; Aharony et al., 2015). Importantly, expression of these fragments containing the polyQ stretch can already give rise to aggregation and the disease phenotype (Ikeda et al., 1996), although it is still not entirely clear why the polyQ fragments display enhanced toxicity when compared to their respective full-length proteins. Cleavage may lead to changes in aggregation propensity, conformation of the protein, localization, and molecular interactions (Matos et al., 2016a). For SBMA, it has been reported that a conformational change exposing the polyQ tract is already sufficient to drive aggregation (Heine et al., 2015) and cleavage might expose the polyQ stretch in a similar way as such a conformational change does. Protein domains that would otherwise prevent, or enhance, the aggregation may be removed, exposing the Q-stretch itself for aggregation. Finally, recognition sites and binding of molecular chaperones could be changed, exemplifying once more the importance of regions outside the polyQ tract in the modulation of aggregation.

For ATXN3, a cleavage product containing the C-terminal fragment from amino acid 221 with the 71Q expansion was found in mice showing the disease phenotype, but rarely in mice not showing the phenotype (Goti et al., 2004). This polypeptide was also found in SCA3 patients (Goti et al., 2004) indicating that fragmentation of the polyQ protein ATXN3 has a strong correlation with disease. Interestingly, while full-length ATXN3 with an expanded polyQ was mostly non-aggregating, co-expression with truncated ATXN3 makes the full-length protein co-localize with the truncated version in perinuclear aggregates (Paulson et al., 1997). More putative cleavage sites in ATXN3 were identified (Haacke et al., 2006; Colomer Gould et al., 2007) and it was shown that caspases are not the sole contributors to the fragmentation of ATXN3, but also the activity of calpains, such as calpain-2, is involved (Simões et al., 2012; Hübener et al., 2013). ATXN3 cleavage and translocation to the nucleus, and thus also aggregation, can be prevented by inhibiting calpains through overexpression of calpastatin in mice (Simões et al., 2012). Conversely, knocking down calpastatin worsened aggregation (Hübener et al., 2013). These data clearly show that under non-stressed conditions *in vivo*, fragmentation is both required and sufficient for aggregation of polyQ containing ATXN3. Similar data has been found for HTT. In almost all studies on HD, a fragment containing the first exon of HTT with the polyQ stretch is being used, since this fragment already gives rise to the HD phenotype. Toxic N-terminal fragments are found to be generated through cleavage by caspases, both in animal models and in patients (Wellington et al., 2002; Sawa et al., 2005; Graham et al., 2006; Maglione et al., 2006). Like in SCA3, fragmentation of HTT is crucial for disease progression, since the HD disease phenotype can be rescued by either mutating the cleavage site of caspase-6 in exon 13 (Graham et al., 2006), genetically ablating caspase-6 (Wong et al., 2015), or pharmacologically inhibiting caspases 1, 3, or 6 (Ona et al., 1999; Chen et al., 2000; Aharony et al., 2015). We have already discussed the ability of certain chaperones to bind to the N17 domain, which is present in the cleaved fragments.

## Post-translational modifications

Post translational modifications (PTMs) like phosphorylation, ubiquitination, and SUMOylation, can affect the aggregation propensity of many polyQ proteins (Humbert et al., 2001; Steffan et al., 2004; Luo et al., 2005; Warby et al., 2005; Menon et al., 2012; Matos et al., 2016b; Figure 2). The transient nature of the PTMs usually indicates differential regulation of proteins and they can provide an interesting extra layer of modulation, possibly influencing all of the above-mentioned features of polyQ aggregation. PTMs can create alternative binding surfaces, affecting the affinity to binding partners like proteases and chaperones, and can lead to conformational changes to expose the Q-stretch. Therefore, either increased or decreased PTMs are associated with aggregation.

For most of the polyQ proteins there are several residues known to be modified (see Figure 2B for PTMs that impact aggregation). For ATXN3 six phosphorylation sites have been described, in the catalytic JD and in the UIMs (Fei et al., 2007; Mueller et al., 2009; Matos et al., 2016b; Figure 2). Phosphorylation of serine (S)340 and S352 in the third UIM did not change aggregation propensity, but shifted the localization of the aggregates from the cytoplasm to the nucleus (Mueller et al., 2009). Phosphorylation of S256 in the second UIM was shown to inhibit the formation of large insoluble polyQ complexes (Fei et al., 2007), and phosphorylation of S12 in the JD also reduces aggregation (Matos et al., 2016b). The protective effect of constitutive phosphorylation of S12 might be dependent on its close proximity to the catalytic sites in the JD, causing hindrance of the intramolecular aggregation. Phosphorylation of HTT on S421 (Humbert et al., 2001) and S434 (Luo et al., 2005), leads to a decrease in polyQ aggregation due to a reduction in caspase-mediated cleavage thus preventing the formation of fragments (Luo et al., 2005; Warby et al., 2009). For ATXN1, S776 is the most studied phosphorylation site since it leads to reduced aggregate formation (Emamian et al., 2003; Orr, 2012). Another interesting PTM on ATXN1 is ubiquitination of K589 in the AXH domain. Mutating this residue leads to reduced degradation and, hence, more aggregation of ATXN1 (Kang et al., 2015), suggesting that PTMs may also affect the degradation of polyQ proteins resulting in a higher concentration of proteins at risk for aggregation.

Chaperone-dependent degradation of still soluble polyQ proteins could therefore be another important aspect in ameliorating disease. Interestingly, the co-chaperone CHIP (C-terminus of Hsp70-interacting protein), an E3 ligase that can interact with and modulate Hsp70 activity (Ballinger et al., 1999; Scheufler et al., 2000), has been implicated as a modulator in many polyQ diseases (Jana et al., 2005; Choi et al., 2007; Gao et al., 2011). CHIP interacts with ATXN1 via the phosphorylated S776 and the phospho-dead S776A mutation reduced this interaction. The CHIP-ATXN1 interaction is likely mediated via Hsp70, since the tetratricopeptide repeat (TPR) domain of CHIP, with which it interacts with Hsp70, is needed for the interaction and for promotion of ATXN1 degradation (Choi et al., 2007). A similar model of CHIP and Hsp70 interaction with HTT and ATXN3 was proposed, although no single modified residue was identified as a recognition site (Jana et al., 2005).

Members of DNAJ family of Hsp70 co-chaperones were also shown to play a role in the PTM dependent degradation of polyQ proteins, like in ATXN3 (Gao et al., 2011). DNAJB1 was identified to suppress aggregate formation of ATXN3 (Chai et al., 1999), but aggregation of the S256A mutant of ATXN3 could not be prevented by DNAJB1 (Fei et al., 2007), it is still unclear whether DNAJB1 has preferential affinity for phosphorylated ATXN3. Interestingly, Hsp70 can prevent S256A aggregation (Fei et al., 2007). Next to DNAJB1, DNAJB2 was found to suppress polyQ protein aggregation via two UIMs that were shown to be crucial for its interaction with K63-linked ubiquitination of HTT (Labbadia et al., 2012). Intriguingly, all the PTMs on HTT are less present in polyQ-expanded HTT, especially in the regions in the brain that are mostly affected, abolishing the possible protective effect of the modifications (Luo et al., 2005; Warby et al., 2005; Aiken et al., 2009). Currently it is unclear whether the drop in modification is causal or a consequence of aggregation.

## Perspectives

The expanded polyQ stretches in the different disease-associated proteins are the determining factor of disease onset and progression in all of the polyglutaminopathies. Above a certain threshold, Q-stretches are prone to aggregate. However, more often than not, the Q-stretch and its aggregation propensities are modulated by secondary events that we categorized here; flanking regions, which have modulating capacity due to intrinsic stability issues, binding of partners (including chaperones), modification by PTMs, and cleavage of the Q-stretch. The examples of molecular interactions described, clearly indicate that polyQ protein aggregation is a multifactorial and likely multistep process that not always has to go through the same sequence of events toward aggregate formation. For example, the intrinsic fibrillogenic behavior of the JD and cleavage of ATXN3 (leading to a fragment not containing the JD) can both trigger aggregation independently. It could very well be that initial aggregation can be triggered via different mechanisms leading to secondary events that stimulate aggregation further. Thus, *in vivo* aggregation of the JD might stimulate ATXN3 cleavage and, vice versa, cleavage might destabilize the JD domain resulting in a fast forward feedback loop of aggregation. Modulating events, together with the unique expression pattern and level of each polyQ protein, could explain the variation in AO between the nine diseases.

Moreover, the modulating events acting on the flanking regions might also explain the variation of AO among patients with a similar Q length within a given polyQ disease. By combining information on Q length (CAG repeat), expression levels of the chaperone DNAJB6, which modulates Q aggregation directly, and the expression levels of chaperones that act on the disease-specific flanking regions, with the PTM and fragmentation status, perhaps a better predication of AO could be made. A strategy targeting chaperones acting on the Q-stretch with those acting on the flanking regions might provide a synergistic approach for delaying AO, benefiting individuals diagnosed with an expanded polyQ tract. There is little information on the factors influencing progression of disease after onset and it would also be of interest to know whether progression of disease is influenced by the same factors that modulate aggregation propensity. If so, these could be used as a therapeutic modality as well.

## Author contributions

EK and ED compiled all the data and contributed equally to this work. EK, ED, LJ, HK, and SB gave intellectual feedback and wrote the manuscript.

### Conflict of interest statement

The authors declare that the research was conducted in the absence of any commercial or financial relationships that could be construed as a potential conflict of interest.
