# Supplementary material for: Chaperones in Polyglutamine Aggregation: Beyond the Q-Stretch
Source: Front Neurosci. 2017 Mar 23;11:145. doi: 10.3389/fnins.2017.00145 (PMC5362620; doi:10.3389/fnins.2017.00145)
Supplement: Supplementary file 1 [file DataSheet1.DOCX]

**References associated with Figures 1 and 2:**

Aharony, I., Ehrnhoefer, D. E., Shruster, A., Qiu, X., Franciosi, S., Hayden, M. R., et al. (2015). A Huntingtin-based peptide inhibitor of caspase-6 provides protection from mutant Huntingtin-induced motor and behavioral deficits. Hum. Mol. Genet. 24, 2604–2614. doi:10.1093/hmg/ddv023.

Almaguer-Mederos, L. E., Falcón, N. S., Almira, Y. R., Zaldivar, Y. G., Almarales, D. C., Góngora, E. M., et al. (2010). Estimation of the age at onset in spinocerebellar ataxia type 2 Cuban patients by survival analysis. Clin. Genet. 78, 169–174. doi:10.1111/j.1399-0004.2009.01358.x.

Almeida, B., Abreu, I. A., Matos, C. A., Fraga, J., Fernandes, S., Macedo, M. G., et al. (2015). SUMOylation of the brain-predominant Ataxin-3 isoform modulates its interaction with p97. Biochim. Biophys. Acta 1852, 1950–9. doi:10.1016/j.bbadis.2015.06.010.

Angeli, S., Shao, J., and Diamond, M. I. (2010). F-actin binding regions on the androgen receptor and huntingtin increase aggregation and alter aggregate characteristics. PLoS One 5. doi:10.1371/journal.pone.0009053.

Berke, S. J. S., Chai, Y., Marrs, G. L., Wen, H., and Paulson, H. L. (2005). Defining the role of ubiquitin-interacting motifs in the polyglutamine disease protein, ataxin-3. J. Biol. Chem. 280, 32026–34. doi:10.1074/jbc.M506084200.

Bonnet, J., Wang, Y. H., Spedale, G., Atkinson, R. A., Romier, C., Hamiche, A., et al. (2010). The structural plasticity of SCA7 domains defines their differential nucleosome-binding properties. EMBO Rep. 11, 612–618. doi:10.1038/embor.2010.98.

Chen, M., Ona, V. O., Li, M., Ferrante, R. J., Fink, K. B., Zhu, S., et al. (2000). Minocycline inhibits caspase-1 and caspase-3 expression and delays mortality in a transgenic mouse model of Huntington disease. Nat. Med. 6, 797–801. doi:10.1038/77528.

Chen, S., Peng, G. H., Wang, X., Smith, A. C., Grote, S. K., Sopher, B. L., et al. (2004). Interference of Crx-dependent transcription by ataxin-7 involves interaction between th glutamine regions and requires the ataxin-7 carboxy-terminal region for nuclear localization. Hum. Mol. Genet. 13, 53–67. doi:10.1093/hmg/ddh005.

Colomer Gould, V. F., Goti, D., Pearce, D., Gonzalez, G. a, Gao, H., Bermudez de Leon, M., et al. (2007). A mutant ataxin-3 fragment results from processing at a site N-terminal to amino acid 190 in brain of Machado-Joseph disease-like transgenic mice. Neurobiol. Dis. 27, 362–9. doi:10.1016/j.nbd.2007.06.005.

Cui, Y., Yang, S., Li, X.-J., and Li, S. (2016). Genetically modified rodent models of SCA17. J. Neurosci. Res. 0. doi:10.1002/jnr.23984.

Darrington, R. S., Butler, R., Leigh, P. N., McPhaul, M. J., and Gallo, J. M. (2002). Ligand-dependent aggregation of polyglutamine-expanded androgen receptor in neuronal cells. Neuroreport 13, 2117–2120. doi:10.1097/00001756-200211150-00025.

Ehrnhoefer, D. E., Sutton, L., and Hayden, M. R. (2011). Small Changes, Big Impact. Neurosci. 17, 475–492. doi:10.1177/1073858410390378.

Ellerby, L. M., Andrusiak, R. L., Wellington, C. L., Hackam, A. S., Propp, S. S., Wood, J. D., et al. (1999). Cleavage of atrophin-1 at caspase site aspartic acid 109 modulates cytotoxicity. J. Biol. Chem. 274, 8730–8736. doi:10.1074/jbc.274.13.8730.

Emamian, E. S., Kaytor, M. D., Duvick, L. A., Zu, T., Tousey, S. K., Zoghbi, H. Y., et al. (2003). Serine 776 of ataxin-1 is critical for polyglutamine-induced disease in SCA1 transgenic mice. Neuron 38, 375–387. doi:10.1016/S0896-6273(03)00258-7.

Fei, E., Jia, N., Zhang, T., Ma, X., Wang, H., Liu, C., et al. (2007). Phosphorylation of ataxin-3 by glycogen synthase kinase 3β at serine 256 regulates the aggregation of ataxin-3. Biochem. Biophys. Res. Commun. 357, 487–492. doi:10.1016/j.bbrc.2007.03.160.

Friedman, M. J., Wang, C. E., Li, X. J., and Li, S. (2008). Polyglutamine expansion reduces the association of TATA-binding protein with DNA and induces DNA binding-independent neurotoxicity. J. Biol. Chem. 283, 8283–8290. doi:10.1074/jbc.M709674200.

Gao, R., Matsuura, T., Coolbaugh, M., Zühlke, C., Nakamura, K., Rasmussen, A., et al. (2008). Instability of expanded CAG/CAA repeats in spinocerebellar ataxia type 17. Eur. J. Hum. Genet. 16, 215–22. doi:10.1038/sj.ejhg.5201954.

Garden, G. a, Libby, R. T., Fu, Y.-H., Kinoshita, Y., Huang, J., Possin, D. E., et al. (2002). Polyglutamine-expanded ataxin-7 promotes non-cell-autonomous purkinje cell degeneration and displays proteolytic cleavage in ataxic transgenic mice. J. Neurosci. 22, 4897–905. doi:22/12/4897 [pii].

Giorgetti, E., and Lieberman, A. P. (2016). Polyglutamine androgen receptor-mediated neuromuscular disease. Cell. Mol. Life Sci. 73, 3991–3999. doi:10.1007/s00018-016-2275-1.

Goti, D., Katzen, S. M., Mez, J., Kurtis, N., Kiluk, J., Ben-Haïem, L., et al. (2004). A mutant ataxin-3 putative-cleavage fragment in brains of Machado-Joseph disease patients and transgenic mice is cytotoxic above a critical concentration. J. Neurosci. 24, 10266–79. doi:10.1523/JNEUROSCI.2734-04.2004.

Graham, R. K., Deng, Y., Slow, E. J., Haigh, B., Bissada, N., Lu, G., et al. (2006). Cleavage at the Caspase-6 Site Is Required for Neuronal Dysfunction and Degeneration Due to Mutant Huntingtin. Cell 125, 1179–1191. doi:10.1016/j.cell.2006.04.026.

Gusella, J. F., and MacDonald, M. E. (2000). Molecular genetics: unmasking polyglutamine triggers in neurodegenerative disease. Nat. Rev. Neurosci. 1, 109–15. doi:10.1038/nrd1077.

Guyenet, S. J., Mookerjee, S. S., Lin, A., Custer, S. K., Chen, S. F., Sopher, B. L., et al. (2015). Proteolytic cleavage of ataxin-7 promotes SCA7 retinal degeneration and neurological dysfunction. Hum. Mol. Genet. 24, 3908–3917. doi:10.1093/hmg/ddv121.

Hübener, J., Weber, J. J., Richter, C., Honold, L., Weiss, A., Murad, F., et al. (2013). Calpain-mediated ataxin-3 cleavage in the molecular pathogenesis of spinocerebellar ataxia type 3 (SCA3). Hum. Mol. Genet. 22, 508–18. doi:10.1093/hmg/dds449.

Ishikawa, K., Watanabe, M., Shoji, S., and Tsuji, S. (1997). Japanese Families with Autosomal Dominant Pure Cerebellar Ataxia\nMap to Chromosome l9pl3.1 -p13.2 and Are Strongly Associated\nwith Mild CAG Expansions in the Spinocerebellar Ataxia Type 6\nGene in Chromosome 19p13.1. Am. Soc. Hum. Genet., 336–346. doi:10.1086/514867.

Jackson, G. R., Salecker, I., Dong, X., Yao, X., Arnheim, N., Faber, P. W., et al. (1998). Polyglutamine-expanded human huntingtin transgenes induce degeneration of Drosophila photoreceptor neurons. Neuron 21, 633–642. doi:10.1016/S0896-6273(00)80573-5.

Janer, A., Werner, A., Takahashi-Fujigasaki, J., Daret, A. l., Fujigasaki, H., Takada, K., et al. (2009). SUMOylation attenuates the aggregation propensity and cellular toxicity of the polyglutamine expanded ataxin-7. Hum. Mol. Genet. 19, 181–195. doi:10.1093/hmg/ddp478.

Jiménez-López, D., and Guzmán, P. (2014). Insights into the Evolution and Domain Structure of Ataxin-2 Proteins Across Eukaryotes. BMC Res. Notes 7, 453. doi:10.1186/1756-0500-7-453.

Jodice, C., Malaspina, P., Persichetti, F., Novelletto, A., Spadaro, M., Giunti, P., et al. (1994). Effect of trinucleotide repeat length and parental sex on phenotypic variation in spinocerebellar ataxia I. Am. J. Hum. Genet. 54, 959–65. Available at: http://www.pubmedcentral.nih.gov/articlerender.fcgi?artid=1918191&tool=pmcentrez&rendertype=abstract.

Kaltenbach, L. S., Romero, E., Becklin, R. R., Chettier, R., Bell, R., Phansalkar, A., et al. (2007). Huntingtin interacting proteins are genetic modifiers of neurodegeneration. PLoS Genet. 3, e82. doi:10.1371/journal.pgen.0030082.

Klement, I. a, Skinner, P. J., Kaytor, M. D., Yi, H., Hersch, S. M., Clark, H. B., et al. (1998). Ataxin-1 nuclear localization and aggregation: role in polyglutamine-induced disease in SCA1 transgenic mice. Cell 95, 41–53. doi: 10.1016/S0092-8674(00)81781-X.

Kordasiewicz, H. B., Thompson, R. M., Clark, H. B., and Gomez, C. M. (2006). C-termini of P/Q-type Ca2+ channel ??1A subunits translocate to nuclei and promote polyglutamine-mediated toxicity. Hum. Mol. Genet. 15, 1587–1599. doi:10.1093/hmg/ddl080.

Kubodera, T., Yokota, T., Ohwada, K., Ishikawa, K., Miura, H., Matsuoka, T., et al. (2003). Proteolytic cleavage and cellular toxicity of the human ??1A calcium channel in spinocerebellar ataxia type 6. Neurosci. Lett. 341, 74–78. doi:10.1016/S0304-3940(03)00156-3.

La Spada, A. R., Fu, Y. H., Sopher, B. L., Libby, R. T., Wang, X., Li, L. Y., et al. (2001). Polyglutamine-expanded ataxin-7 antagonizes CRX function and induces cone-rod dystrophy in a mouse model of SCA7. Neuron 31, 913–927. doi:10.1016/S0896-6273(01)00422-6.

LaFevre-Bernt, M. A., and Ellerby, L. M. (2003). Kennedy’s disease: Phosphorylation of the polyglutamine-expanded form of androgen receptor regulates its clevage by caspase-3 and enhances cell death. J. Biol. Chem. 278, 34918–34924. doi:10.1074/jbc.M302841200.

Lam, Y. C., Bowman, A. B., Jafar-Nejad, P., Lim, J., Richman, R., Fryer, J. D., et al. (2006). ATAXIN-1 interacts with the repressor Capicua in its native complex to cause SCA1 neuropathology. Cell 127, 1335–47. doi:10.1016/j.cell.2006.11.038.

Lin, H. K., Yeh, S., Kang, H. Y., and Chang, C. (2001). Akt suppresses androgen-induced apoptosis by phosphorylating and inhibiting androgen receptor. Proc. Natl. Acad. Sci. U. S. A. 98, 7200–5. doi:10.1073/pnas.121173298.

Lund, a, Udd, B., Juvonen, V., Andersen, P. M., Cederquist, K., Davis, M., et al. (2001). Multiple founder effects in spinal and bulbar muscular atrophy (SBMA, Kennedy disease) around the world. Eur. J. Hum. Genet. 9, 431–436. doi:10.1038/sj.ejhg.5200656.

Maglione, V., Cannella, M., Gradini, R., Cislaghi, G., and Squitieri, F. (2006). Huntingtin fragmentation and increased caspase 3, 8 and 9 activities in lymphoblasts with heterozygous and homozygous Huntington’s disease mutation. Mech. Ageing Dev. 127, 213–216. doi:10.1016/j.mad.2005.09.011.

Mangiarini, L., Sathasivam, K., Seller, M., Cozens, B., Harper, A., Hetherington, C., et al. (1996). Exon I of the HD gene with an expanded CAG repeat is sufficient to cause a progressive neurological phenotype in transgenic mice. Cell 87, 493–506. doi:10.1016/S0092-8674(00)81369-0.

Martin, D. D. O., Heit, R. J., Yap, M. C., Davidson, M. W., Hayden, M. R., and Berthiaume, L. G. (2014). Identification of a post-translationally myristoylated autophagy-inducing domain released by caspase cleavage of huntingtin. Hum. Mol. Genet. 23, 3166–3179. doi:10.1093/hmg/ddu027.

Martindale, D., Hackam, A., Wieczorek, A., Ellerby, L., Wellington, C., McCutcheon, K., et al. (1998). Length of huntingtin and its polyglutamine tract influences localization and frequency of intracellular aggregates. Nat. Genet. 18, 150–4. doi:10.1038/ng0298-150.

Matos, C. A., Nóbrega, C., Louros, S. R., Almeida, B., Ferreiro, E., Valero, J., et al. (2016). Ataxin-3 phosphorylation decreases neuronal defects in spinocerebellar ataxia type 3 models. J. Cell Biol. 212, 465–480. doi:10.1083/jcb.201506025.

Matsuyama, Z., Kawakami, H., Maruyama, H., Izumi, Y., Komure, O., Udaka, F., et al. (1997). Molecular features of the CAG repeats of spinocerebellar ataxia 6 (SCA6). Hum. Mol. Genet. 6, 1283–1287. doi:10.1093/hmg/6.8.1283.

Miyashita, T., Okamura-Oho, Y., Mito, Y., Nagafuchi, S., and Yamada, M. (1997). Dentatorubral pallidoluysian atrophy (DRPLA) protein is cleaved by caspase-3 during apoptosis. J. Biol. Chem. 272, 29238–29242. doi:10.1074/jbc.272.46.29238.

Montie, H. L., Pestell, R. G., and Merry, D. E. (2011). SIRT1 modulates aggregation and toxicity through deacetylation of the androgen receptor in cell models of SBMA. J Neurosci 31, 17425–17436. doi:10.1523/jneurosci.3958-11.2011.

Mookerjee, S., Papanikolaou, T., Guyenet, S. J., Sampath, V., Lin, A., Vitelli, C., et al. (2009). Posttranslational modification of ataxin-7 at lysine 257 prevents autophagy-mediated turnover of an N-terminal caspase-7 cleavage fragment. J. Neurosci. 29, 15134–15144. doi:10.1523/JNEUROSCI.4720-09.2009.

Mueller, T., Breuer, P., Schmitt, I., Walter, J., Evert, B. O., and Wüllner, U. (2009). CK2-dependent phosphorylation determines cellular localization and stability of ataxin-3. Hum. Mol. Genet. 18, 3334–43. doi:10.1093/hmg/ddp274.

Mukherjee, S., Thomas, M., Dadgar, N., Lieberman, A. P., and Iñiguez-Lluhi, J. A. (2009). Small ubiquitin-like modifier (SUMO) modification of the androgen receptor attenuates polyglutamine-mediated aggregation. J. Biol. Chem. 284, 21296–21306. doi:10.1074/jbc.M109.011494.

Nakamura, K. (2001). SCA17, a novel autosomal dominant cerebellar ataxia caused by an expanded polyglutamine in TATA-binding protein. Hum. Mol. Genet. 10, 1441–1448. doi:10.1093/hmg/10.14.1441.

Nozaki, K., Onodera, O., Takano, H., and Tsuji, S. (2001). Amino acid sequences flanking polyglutamine stretches influence their potential for aggregate formation. Neuroreport 12, 3357–3364. doi:10.1097/00001756-200110290-00042.

Nucifora, F. C., Ellerby, L. M., Wellington, C. L., Wood, J. D., Herring, W. J., Sawa, A., et al. (2003). Nuclear localization of a non-caspase truncation product of atrophin-1, with an expanded polyglutamine repeat, increases cellular toxicity. J. Biol. Chem. 278, 13047–13055. doi:10.1074/jbc.M211224200.

Okamura-Oho, Y., Miyashita, T., Nagao, K., Shima, S., Ogata, Y., Katada, T., et al. (2003). Dentatorubral-pallidoluysian atrophy protein is phosphorylated by c-Jun NH2-terminal kinase. Hum. Mol. Genet. 12, 1535–1542. doi:10.1093/hmg/ddg168.

Palazzolo, I., Burnett, B. G., Young, J. E., Brenne, P. L., La Spada, A. R., Fischbeck, K. H., et al. (2007). Akt blocks ligand binding and protects against expanded polyglutamine androgen receptor toxicity. Hum. Mol. Genet. 16, 1593–1603. doi:10.1093/hmg/ddm109.

Polanco, M. J., Parodi, S., Piol, D., Stack, C., Chivet, M., Contestabile, A., et al. (2016). Adenylyl cyclase activating polypeptide reduces phosphorylation and toxicity of the polyglutamine-expanded androgen receptor in spinobulbar muscular atrophy. 181. doi:10.1126/scitranslmed.aaf9526.

Ranum, L. P., Chung, M. Y., Banfi, S., Bryer, a, Schut, L. J., Ramesar, R., et al. (1994). Molecular and clinical correlations in spinocerebellar ataxia type I: evidence for familial effects on the age at onset. Am. J. Hum. Genet. 55, 244–52. Available at: http://www.pubmedcentral.nih.gov/articlerender.fcgi?artid=1918367&tool=pmcentrez&rendertype=abstract.

Riley, B. E., Zoghbi, H. Y., and Orr, H. T. (2005). SUMOylation of the polyglutamine repeat protein, ataxin-1, is dependent on a functional nuclear localization signal. J. Biol. Chem. 280, 21942–21948. doi:10.1074/jbc.M501677200.

Rosenblatt, A., Brinkman, R. R., Liang, K. Y., Almqvist, E. W., Margolis, R. L., Huang, C. Y., et al. (2001). Familial influence on age of onset among siblings with huntington disease. Am. J. Med. Genet. - Neuropsychiatr. Genet. 105, 399–403. doi:10.1002/ajmg.1400.

Saute, J. A. M., and Jardim, L. B. (2015). Machado Joseph disease: clinical and genetic aspects, and current treatment. Expert Opin. Orphan Drugs, 1–19. doi:10.1517/21678707.2015.1025747.

Sawa, A., Nagata, E., Sutcliffe, S., Dulloor, P., Cascio, M. B., Ozeki, Y., et al. (2005). Huntingtin is cleaved by caspases in the cytoplasm and translocated to the nucleus via perinuclear sites in Huntington’s disease patient lymphoblasts. Neurobiol. Dis. 20, 267–274. doi:10.1016/j.nbd.2005.02.013.

Scaramuzzino, C., Casci, I., Parodi, S., Lievens, P. M. J., Polanco, M. J., Milioto, C., et al. (2015). Protein Arginine Methyltransferase 6 Enhances Polyglutamine-Expanded Androgen Receptor Function and Toxicity in Spinal and Bulbar Muscular Atrophy. Neuron 85, 88–101. doi:10.1016/j.neuron.2014.12.031.

Shatunov, A., Fridman, E. A., Pagan, F. I., Leib, J., Singleton, A., Hallett, M., et al. (2004). Small de novo duplication in the repeat region of the TATA-box-binding protein gene manifest with a phenotype similar to variant Creutzfeldt-Jakob disease. Clin. Genet. 66, 496–501. doi:10.1111/j.1399-0004.2004.00356.x.

Silveira, I., Miranda, C., Guimarães, L., Moreira, M.-C., Alonso, I., Mendonça, P., et al. (2002). Trinucleotide repeats in 202 families with ataxia: a small expanded (CAG)n allele at the SCA17 locus. Arch. Neurol. 59, 623–9. doi: 10.1001/archneur.59.4.623.

Sperfeld, A. D., Karitzky, J., Brummer, D., Schreiber, H., Häussler, J., Ludolph, A. C., et al. (2002). X-linked bulbospinal neuronopathy: Kennedy disease. Arch. Neurol. 59, 1921–6. doi: 10.1001/archneur.59.12.1921.

Steffan, J. S., Agrawal, N., Pallos, J., Rockabrand, E., Trotman, L. C., Slepko, N., et al. (2004). SUMO modification of Huntingtin and Huntington’s disease pathology. Science 304, 100–104. doi:10.1126/science.1092194.

Suzuki, Y., Nakayama, K., Hashimoto, N., and Yazawa, I. (2010). Proteolytic processing regulates pathological accumulation in dentatorubral-pallidoluysian atrophy. FEBS J. 277, 4873–4887. doi:10.1111/j.1742-4658.2010.07893.x.

Taylor, J., Grote, S. K., Xia, J., Vandelft, M., Graczyk, J., Ellerby, L. M., et al. (2006). Ataxin-7 can export from the nucleus via a conserved exportin-dependent signal. J. Biol. Chem. 281, 2730–2739. doi:10.1074/jbc.M506751200.

Todi, S. V, Scaglione, K. M., Blount, J. R., Basrur, V., Conlon, K. P., Pastore, A., et al. (2010). Activity and cellular functions of the deubiquitinating enzyme and polyglutamine disease protein ataxin-3 are regulated by ubiquitination at lysine 117. J. Biol. Chem. 285, 39303–13. doi:10.1074/jbc.M110.181610.

Tsuda, H., Jafar-Nejad, H., Patel, A. J., Sun, Y., Chen, H. K., Rose, M. F., et al. (2005). The AXH domain of ataxin-1 mediates neurodegeneration through its interaction with Gfi-1/senseless proteins. Cell 122, 633–644. doi:10.1016/j.cell.2005.06.012.

van de Warrenburg, B. P. C., Hendriks, H., Dürr, A., van Zuijlen, M. C. a, Stevanin, G., Camuzat, A., et al. (2005). Age at onset variance analysis in spinocerebellar ataxias: a study in a Dutch-French cohort. Ann. Neurol. 57, 505–12. doi:10.1002/ana.20424.

Vinton, A., Fahey, M. C., O’Brien, T. J., Shaw, J., Storey, E., Gardner, R. J. M., et al. (2005). Dentatorubral-pallidoluysian atrophy in three generations, with clinical courses from nearly asymptomatic elderly to severe juvenile, in an Australian family of Macedonian descent. Am. J. Med. Genet. 136 A, 201–204. doi:10.1002/ajmg.a.30355.

Warby, S. C., Chan, E. Y., Metzler, M., Gan, L., Singaraja, R. R., Crocker, S. F., et al. (2005). Huntingtin phosphorylation on serine 421 is significantly reduced in the striatum and by polyglutamine expansion in vivo. Hum. Mol. Genet. 14, 1569–1577. doi:10.1093/hmg/ddi165.

Wellington, C. L., Ellerby, L. M., Gutekunst, C.-A., Rogers, D., Warby, S., Graham, R. K., et al. (2002). Caspase cleavage of mutant huntingtin precedes neurodegeneration in Huntington’s disease. J. Cell Biol. 22, 749–759. doi:10.1038/35096019.

Wellington, C. L., Ellerby, L. M., Hackam, A. S., Margolis, R. L., Trifiro, M. A., Singaraja, R., et al. (1998). Caspase cleavage of gene products associated with triplet expansion disorders generates truncated fragments containing the polyglutamine tract. J. Biol. Chem. 273, 9158–67. doi: 10.1074/jbc.273.15.9158.

Wexler, N. S., Lorimer, J., Porter, J., Gomez, F., Moskowitz, C., Shackell, E., et al. (2004). Venezuelan kindreds reveal that genetic and environmental factors modulate Huntington’s disease age of onset. Proc. Natl. Acad. Sci. U. S. A. 101, 3498–503. doi:10.1073/pnas.0308679101.

Wong, B. K. Y., Ehrnhoefer, D. E., Graham, R. K., Martin, D. D. O., Ladha, S., Uribe, V., et al. (2015). Partial rescue of some features of Huntington Disease in the genetic absence of caspase-6 in YAC128 mice. Neurobiol. Dis. 76, 24–36. doi:10.1016/j.nbd.2014.12.030.

Yang, S., Li, X.-J., and Li, S. (2016). Molecular mechanisms underlying Spinocerebellar Ataxia 17 (SCA17) pathogenesis. Rare Dis. 4, e1223580. doi:10.1080/21675511.2016.1223580.

Young, J. E., Garden, G. A., Martinez, R. A., Tanaka, F., Miguel Sandoval, C., Smith, A. C., et al. (2009). Polyglutamine-Expanded Androgen Receptor Truncation Fragments Activate a Bax-Dependent Apoptotic Cascade Mediated by DP5/Hrk. J. Neurosci. 29, 1987–1997. doi:10.1523/JNEUROSCI.4072-08.2009.

Young, J. E., Gouw, L., Propp, S., Sopher, B. L., Taylor, J., Lin, A., et al. (2007). Proteolytic cleavage of ataxin-7 by caspase-7 modulates cellular toxicity and transcriptional dysregulation. J. Biol. Chem. 282, 30150–30160. doi:10.1074/jbc.M705265200.

Yvert, G., Lindenberg, K. S., Picaud, S., Landwehrmeyer, G. B., Sahel, J. a, and Mandel, J. L. (2000). Expanded polyglutamines induce neurodegeneration and trans-neuronal alterations in cerebellum and retina of SCA7 transgenic mice. Hum. Mol. Genet. 9, 2491–2506. doi:10.1093/hmg/9.17.2491.

Zboray, L., Pluciennik, A., Curtis, D., Liu, Y., Berman-Booty, L. D., Orr, C., et al. (2015). Preventing the Androgen Receptor N/C Interaction Delays Disease Onset in a Mouse Model of SBMA. Cell Rep. 13, 2312–2323. doi:10.1016/j.celrep.2015.11.019.

Zhou, Y.-F., Liao, S.-S., Luo, Y.-Y., Tang, J.-G., Wang, J.-L., Lei, L.-F., et al. (2013). SUMO-1 modification on K166 of polyQ-expanded ataxin-3 strengthens its stability and increases its cytotoxicity. PLoS One 8, e54214. doi:10.1371/journal.pone.0054214.
